# Time-lapse imaging of cortical projection neuron migration in mice using mosaic analysis with double markers

**DOI:** 10.1016/j.xpro.2023.102795

**Published:** 2024-01-01

**Authors:** Andi H. Hansen, Simon Hippenmeyer

**Affiliations:** 1 Institute of Science and Technology Austria (ISTA), Am Campus 1, 3400 Klosterneuburg, Austria

**Keywords:** Single Cell, Genetics, Microscopy

## Abstract

Mosaic analysis with double markers (MADM) technology enables the sparse labeling of genetically defined neurons. We present a protocol for time-lapse imaging of cortical projection neuron migration in mice using MADM. We describe steps for the isolation, culturing, and 4D imaging of neuronal dynamics in MADM-labeled brain tissue. While this protocol is compatible with other single-cell labeling methods, the MADM approach provides a genetic platform for the functional assessment of cell-autonomous candidate gene function and the relative contribution of non-cell-autonomous effects.

For complete details on the use and execution of this protocol, please refer to Hansen et al. (2022),^1^ Contreras et al. (2021),^2^ and Amberg and Hippenmeyer (2021).^3^

## Highlights

• Workflow for the preparation of cultured murine MADM brain slices
• Guidance for time-lapse imaging of live MADM brain slices
• Correction of non-linear local drift in time-lapse images
• Analysis of neuronal migration trajectories acquired from time-lapse imaging

## Abstract (editor highlights)

Publisher’s note: Undertaking any experimental protocol requires adherence to local institutional guidelines for laboratory safety and ethics.

## Graphical abstract

**Figure:**
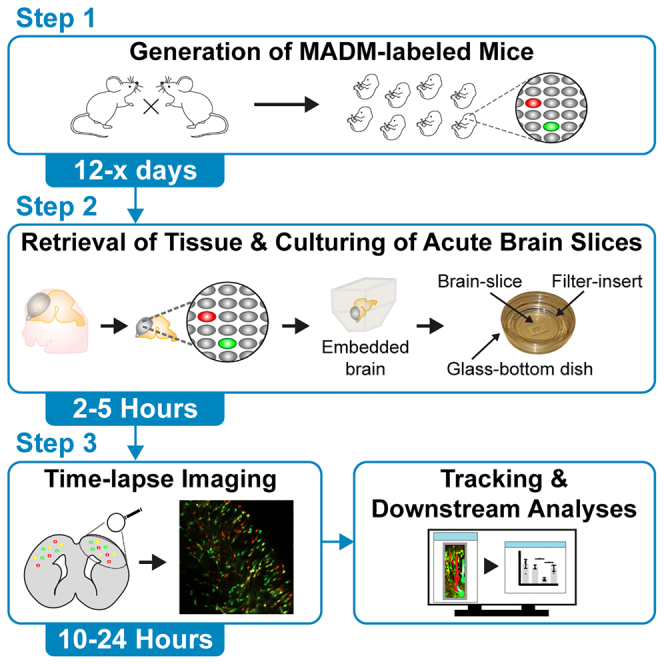

## Before you begin

### Background

This protocol describes 4D time-lapse imaging of Mosaic Analysis with Double Markers (MADM)-labeled brain tissue. MADM is a genetic technology that allows the introduction of a homozygous mutation of choice with unprecedented single cell resolution in a defined genetic lineage with concomitant fluorescent labeling.^2^^,^^4^ As MADM technology allows for the analysis of sparse genetic mosaic versus global/whole tissue ablation of a candidate gene with single cell resolution it provides a unique genetic tool to investigate cell-autonomous gene functions and the relative contribution of non-cell-autonomous effects, which can be quantitatively analyzed by time-lapse imaging. Other techniques for sparse labeling, such as viral-induced, *in utero* electroporation, mT/mG mice, are readily available and could be utilized in conjunction with this protocol. Several potential sparse labeling techniques have been reviewed elsewhere^5^^,^^6^^,^^7^

In this protocol, we present an experimental pipeline for the isolation, culturing and 4D imaging of MADM-labeled brain tissue *in situ* that allows for the analysis of neuronal migration dynamics. Please refer to^1^ for further information. For details on the MADM technology, please refer to.^2^^,^^3^^,^^4^^,^^8^

See Contreras et al. and Amberg et al. for details on choosing and breeding the MADM line of choice.^2^^,^^3^

### Prepare dissection equipment (on the day of experiment)

**Timing: 15 min**

• Clean all dissection tools (2 curved forceps, large and small scissors) with 70% ethanol.
• Prepare 24-well plates filled with ice-cold PBS to collect embryos (store plate on ice).
• Prepare 24 1.5 mL Eppendorf tubes to collect tissue for genotyping.
• Prepare respective genotyping reagents.

### Prepare slice-culture dishes (on the day of experiment)

**Timing: 15 min**

• Fill each well (35 mm glass-bottom dish or 6-well glass-bottom plate) with 1.5 mL of culture medium (see culture medium).
• Place a cell culture insert in each well/dish. Use high edge filter-inserts as they are heavier and therefore not so prone to move.
• Place in incubator (37°C, 5% CO2) until needed.

### Vibratome sectioning preparations

• Keep the vibratome cutting chamber and cutting stage at ‒20°C before use.

***Note:*** For MADM to work, the presence of a Cre recombinase, active in stem or progenitor cells of your tissue of interest, is required. In other words, any Cre-driver can be used, provided Cre is expressed in dividing stem and progenitor cells, to target cell types and lineages of interest.

### Institutional permissions

Institutional and governmental permission and oversight information for the animal study should be obtained. In this study, experimental procedures were discussed and approved by the institutional ethics and animal welfare committees at IST Austria in accordance with good scientific practice guidelines and national legislation (license number: BMWF-66.018/0007-II/3b/2012 and BMWFW-66.018/0006-WF/V/3b/2017).

## Key resources table

**Table:** 

REAGENT or RESOURCE	SOURCE	IDENTIFIER
**Chemicals, peptides, and recombinant proteins**		

10X PBS	Thermo Scientific	Cat# 70011
Ethanol	Honeywell	Cat# 24194
Penicillin-Streptomycin (5,000 U/mL) (PenStrep)	Gibco	Cat# 15070063
DMEM/F12, no phenol red	Gibco	Cat# 21041025
N-2 supplement (100X)	Gibco	Cat# 17502001
D-(+)-glucose	Sigma-Aldrich	N/A
Low-melting agarose	Fisher BioReagents	Cat# 10377033
Cyanoacrylat-based super glue (standard superglue)	N/A	N/A

**Experimental models: Organisms/strains**		

*Mus musculus*: MADM-11-GT Age: 2 months to 1 year; both sexes Developmental stages: E14/E16	The Jackson Laboratory	RRID: IMSR_JAX:013749
*Mus musculus*: MADM-11-TG Age: 2 months to 1 year; both sexes Developmental stages: E14/E16	The Jackson Laboratory	RRID: IMSR_JAX:013751
*Mus musculus*: MADM-5-GT Age: 2 months to 1 year; both sexes Developmental stages: E14/E16	EMMA	RRID: IMSR_EM:14692
*Mus musculus*: MADM-5-TG Age: 2 months to– 1 year; both sexes Developmental stages: E14/E16	EMMA	RRID: IMSR_EM:14693
*Mus musculus*: *Emx1*-Cre Age: 2 months to 1 year; both sexes Developmental stages: E14/E16	The Jackson Laboratory	RRID: IMSR_JAX:005628

**Software and algorithms**		

ZEN blue	Zeiss	http://www.zeiss.com/microscopy/en_us/products/microscope-software/zen.html#introduction
ImageJ 1.52n	N/A	https://imagej.net/
TrackMate v3.8.0	N/A	https://imagej.net/TrackMate
GraphPad Prism	GraphPad	https://www.graphpad.com/scientific-software/prism/
Python 3.9	Anaconda	https://www.anaconda.com/products/distribution
Scripts and code used in this protocol	Hansen et al.^3^	http://github.com/hippenmeyerlab/undrift https://doi.org/10.5281/zenodo.10183022 (https://zenodo.org/records/10183022)

**Other**		

Embedding molds for coronal brain sections	Polysciences, Inc.	Cat# 18986-1
Gas incubation system for CO_2_ and O_2_	ibidi	Cat# 11922
FoilCover-Set for multiplates	PeCon	Cat# 0430.100
Glass-bottom dishes	MatTek	Cat# P06G-1.5-10-F
Millicell culture inserts (high edge)	Millipore	Cat# PICM03050
6-well plates	TPP	Cat# 92406
24-well plates	TPP	Cat# 92424
Flask filters 500 mL	TPP	Cat# 99505
Microfuge tubes 1.5 mL	Thermo Fisher Scientific	Cat# AM12450
50 mL centrifuge tubes	Sarstedt	Cat# 62.547.254
15 mL centrifuge tubes	Sarstedt	Cat# 62.554.502
0.2 micron filter	Nalgene	Cat# 194-2520
Petri dish	Thermo Scientific	Cat# NC9565080
Dissection tools	FST	Various, depending on the specific needs of the experiment
Vibratome VT 1200S	Leica Biosystems	N/A
Buffer tray	Leica Biosystems	N/A
Specimen disc	Leica Biosystems	N/A
Gillette Super Silver/Personna double edge razor blades	Gillette/Personna	N/A
Robu micro-filter-candle	Robu Glasfilter-Geräte GmbH	https://www.robuglas.com/
LSM 800 inverted confocal	Zeiss	N/A

## Materials and equipment

### Essential buffers for tissue harvesting and culturing (prepare fresh on the day of the experiment):

**Timing: 45 min**

**Table:** Artificial Cerebrospinal Fluid (ACSF) – Prepare fresh on the day of experiment

Reagent	Final concentration	Amount
NaCl	125 mM	7,305 *g*
NaHCO_3_	25 mM	2,100 *g*
KCl	2,5 mM	0,186 *g*
NaH_2_PO_4_	1,25 mM	0,172 *g*
Glucose	25 mM	4,954 *g*
CaCl_2_	2 mM	0,294 *g*
MgCl_2_	1 mM	0,203 *g*
ddH_2_O	NA	1000 mL
**Total**		**1 L**

Optimally, the ACSF solution should be made fresh on the day according to above table. However, to save time, stock solutions can be prepared as described below.

**Table:** AS1 stock solution

Reagent	Final concentration	Amount
NaCl	1.25M	73,05 *g*
KCl	25 mM	1,864 *g*
NaH_2_PO_4_	12.5 mM	1,725 *g*
CaCl_2_	20 mM	2,94 *g*
MgCl_2_	10 mM	2,03 *g*
ddH_2_O	NA	1000 mL
**Total**		**1 L**

**Table:** AS2 stock solution

Reagent	Final concentration	Amount
NaHCO_3_	250 mM	21,003 *g*
ddH_2_O	NA	1000 mL
**Total**		**1 L**

**Table:** D-(+)-Glucose stock solution

Reagent	Final concentration	Amount
Glucose	2.5 M	495.4 *g*
ddH_2_O	NA	1000 mL
**Total**		**1 L**

• prepare 10X AS1 solution (1 L): dilute NaCl 73.05 *g*, KCl 1.864 *g*, NaH_2_PO_4_ 1.725 *g*, CaCl_2_, 2.94 *g*, MgCl_2_ 2.03 *g* in distilled water to 1 L and store at 4°C until needed (store max. 3 months).
• Prepare 10X AS2 Solution (1 L): NaHCO_3_ 21.003 *g* diluted in distilled water to 1 L and store at 4°C (store max. 3 months).
• Prepare D-(+)-Glucose 49.54% solution (Sigma-Aldrich): 495.4 *g* in 1 L ddH_2_O and store at 4°C until needed (store max. 3 months).

Final solution.

• Mix 100 mL AS1 solution + 100 mL AS2 solution + 10 mL 49.54% D-(+)-glucose solution and add ddH_2_O to 1 L (the solution can be kept at 4°C up to 3–4 weeks).
• Oxygenate the solution with carbogen immediately (Using a Robu micro-filter-candle) and continuously throughout the experiment and check that the oxygenated ACSF has pH 7.4.

***Note:*** The osmolality should be approx. 316 mOsm/L. It is however not necessary to determine this for keeping the slices viable as they are cultured immediately after being sliced. However, if one wants to measure the osmolality, it would require an osmolality meter.

**Table:** 4% Low-Melting Agarose (LMA) in ACSF

Reagent	Final concentration	Amount
Low-Melting Agarose	4%	2 *g*
ACSF	N/A	50 mL
**Total**	**4%**	**50 mL**

Detail for preparation of LMA:

• Prepare needed amount of 4% low-melting agarose in ACSF (example: 2 *g* LMA in 50 mL ACSF).
• Vortex and slowly melt in a microwave until all agarose has melted.
• Store in a water bath at 37°C.

**Table:** Culture medium:

Reagent	Final concentration	Amount
100X N2 supplement	1%	1 mL
Penicillin-Streptomycin (5,000 U/mL) (PenStrep)	1%	1 mL
F12/DMEM (no phenol red)	N/A	100 mL
**Total**		**100 mL**

Details for culture medium preparation:

• Dilute 1 mL 100X N2 supplement and 1 mL PenStrep in 100 mL F12/DMEM (no phenol red).
• Sterilize by filtering using a 0.22 μm flask filter into a 250 mL flask.
• Store at 37°C until use.

***Note:*** One can make aliquots by sterile filtering the media into suitable containers and freeze them for later experiments.

**Table:** 1X PBS:

Reagent	Final concentration	Amount
10X PBS	1X	100 mL
ddH_2_O	N/A	900 mL
**Total**	**1X**	**1000 mL**

Keep on ice.

## Step-by-step method details

### Breeding of experimental MADM mice for the generation of genetic mosaic tissue

**Timing: 12-x days (depending on desired developmental time point)**

See Contreras et al. 2021 and Amberg et al. 2021 for details on choosing and breeding the MADM line of choice.

The embryonic age used in this protocol was E14 and E16; however, other developmental stages can be used as desired.

### Retrieval of tissue and culturing of acute brain-tissue slices

**Timing: 2–5 h (depending on the genotyping protocol)**

Here, we will describe the process of retrieving the mouse brain tissue. Depending on the gene of interest and the Cre-driver that is used in the experimental MADM paradigm, use the appropriate genotyping protocol to identify the embryos with the correct genotype. Time is of the essence when dealing with living tissue, so minimizing the time between dissection and imaging is highly recommended to ensure tissue viability.

**Figure 1:**
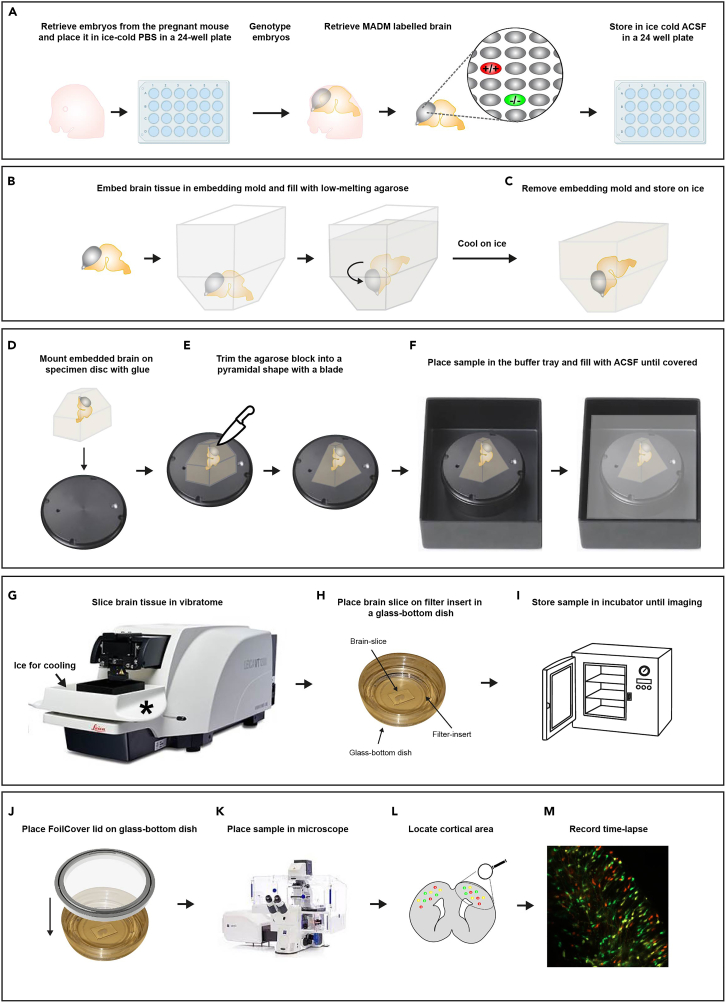
Time-lapse imaging of MADM-labeled tissue (A) Retrieve embryos from the pregnant mouse at the desired developmental stage and genotype to select the brains with MADM labeling. Extracted brains should be stored in ice-cold ACSF until further use. (B) Embed the MADM-labeled brain in an embedding mold and fill with low-melting agarose and position it with the olfactory bulbs facing down. (C) Cool on ice to stiffen the agarose where after the embedding mold can be removed. (D) Mount the embedded brain on the specimen disc with glue. (E) Trim the agarose block into a pyramidal shape. (F) Place the sample in the buffer tray and fill with ice-cold oxygenated ACSF until covered. (G) Place the prepared buffer tray with the mounted specimen in the ice-cooling tray of the vibratome and start slicing the brain. (H) Place the freshly sliced brain tissue on the prepared filter-inserts mounted in a glass-bottom dish. (I) Store the sample in an incubator until imaging. (J) Place the FoilCover lid on the glass-bottom dish containing the sample before placing it in the microscope with an incubator. (K) Place the sample in the microscope and make sure that the climate within the microscope incubator is as desired. (L) Locate the desired region of interest and start imaging with the desired settings. (M) Record the time-lapse over desired length of time.
3. Place each embryo in a 24-well plate containing ice cold 1X PBS and place a small piece of embryonic tissue in a 1.5 mL Eppendorf tube for genotyping.
4. Decapitate the embryo and dissect out the brain in cold ACSF.
5. Place the retrieved brain into ice-cold pre-oxygenated ACSF in another 24-well plate until genotyping has finished.

***Note:*** The genotyping protocol we used typically took 3 hours to complete. If using MADM, one can also check for fluorescent signal using e.g. a fluorescent stereo-microscope to verify correct genotype and/or appropriate fluorescent signal before proceeding to the next step. If a time-consuming genotyping protocol is required, please see problem and solution 1 (see Troubleshooting Problem 1).

1. Sacrifice pregnant dam by cervical dislocation and remove embryos by c-section.
2. Retrieve embryos and isolate brain (Figure 1A).

6. Embedding the brain (Figure 1B).
7. Carefully remove any excess ACSF and place embryonic brain into an embedding mold.
8. Pour 4% low-melting agarose solution into the embedding mold to cover the brain. You may need to gently swirl the brain around in the agarose to dilute any ACSF that was transferred along with the brain to ensure the agarose can bind to the tissue.
9. Orient the brain as needed and keep on ice to let the agarose harden.

### Vibratome sectioning

10. Remove the embedding mold from the agarose-embedded brain (Figure 1C).
11. Dry the agarose-block with embedded brain on the wider bottom by placing it on tissue paper.
12. Place a few drops of super glue on the vibratome specimen disc (Figure 1D) and position the dried agarose block on the glue as needed and let dry for 10 s (coronal sectioning, olfactory bulbs facing upwards).
13. Trim the agarose block with a blade in a pyramidal shape, base of the block wider than the top, making sure the olfactory bulbs are directed to the top of the pyramid shape (Figure 1E). Make sure to keep a few millimeters of agarose around the brain.
14. Place the specimen disc with the trimmed agarose block containing the brain in the buffer tray.
15. Immediately pour ice-cooled oxygenated ACSF in the buffer tray to completely cover the embedded brain and oxygenate the chamber with carbogen (Figure 1C).
16. Fill the vibratome ice-chamber with ice surrounding the buffer tray (Figure 1G).
17. Section the embedded brain coronally at 300 μm (Vibratome setting: 0.4 mm/s, amplitude 1.00 mm).
18. As soon as the slice has been cut within the brain region of interest, grab the slice carefully with a forceps (grabbing the surrounding agarose, if possible) and transfer it directly to the millicell insert in the prepared 35 mm glass-bottom culture dish (Figure 1H) (see Troubleshooting Problem 2).
19. Add culture media on the filter-insert so that the brain slice is covered but make sure the slices does not float and stays positioned on the filter-insert (Figure 1H). Slices can be moved in the desired position by slowly pushing on the edge of the slice while on the filter-insert (see Troubleshooting Problem 3).
20. Place the glass-bottom dish with positioned brain slices in an incubator (37°C, 5% CO2) until ready for the microscope (Figure 1I). We advise readers to commence imaging of the tissue promptly as the disintegration and decrease of viability of the brain slices can occur (see Troubleshooting Problem 4 & 5).

***Note:*** 6-well glass-bottom dishes can be used instead of single 35 mm dishes when imaging several individual brains. Make sure the microscope stage has inserts which can hold either 35 mm or 6-well dishes.

***Note:*** Prenatal brains can stay on ice for some time, whereas postnatal and adult brain tissue should be sectioned immediately. Several brain slices from the same animal can be placed on the same filter-insert to increase the number of slices, which can be imaged.

***Note:*** Buffer trays can be kept in the freezer at ‒20 degrees to ensure a cold environment for longer time when in use.

### Time-lapse imaging of live brain slices

**Timing: 10–24 h depending on the desired imaging time**

Here we will describe a setup using an inverted Zeiss LSM800 fitted with a Plan-Apochromat 10x/0.45, WD = 2.1 mm objective and equipped with a heating chamber and an ibidi stage-top incubator chamber & gas mixer. Time-lapse images were collected on a PC running Zeiss ZEN Blue software. See Methods videos S1, S2, S3, and S4 for desired outcome examples related to step 31.

21. Start the microscope incubator with gas-mixer and set to 37°C, 5% CO2 and let it reach the set values.
22. Mount the FoilCover (PeCon) lid on the glass-bottom-dish to allow gas exchange but reduce the evaporation of the medium fluid (Figure 1J).
23. Place the glass-bottom dish with FoilCover lid holding the prepared brain slices in the microscope stage (Figure 1K).
24. Let the sample acclimatize for ∼45 min to reduce the risk of sample drift.
25. Select the needed laser lines and filters. For MADM tissue, record in two distinct channels with excitation/emission wavelengths at 488/509 GFP and 554/581 nm for tdTomato.
26. Select appropriate objective (we recommend 10x objective for neuronal migration studies), scanning speed and resolution appropriate to the needs of your experiments.
27. Adjust laser intensity and gain for each channel.
28. Select the region of interest for each sample and adjust the microscope accordingly in xyz directions (Figure 1L).
29. Record a time-lapse for each sample (Figure 1M) e.g., for 15 h with a framerate of 15 min recorded unidirectionally at 7 Z-positions with 5 μm spacing between z-planes.
30. Save recorded time-lapse images as ∗.czi files.
31. Export time-lapse images as ∗.tiff files for analysis.

***Note:*** When samples for imaging are placed on filter-inserts, i.e. placed away from the bottom of the glass-bottom dish, it is important to make sure to use a microscope objective with a long working distance e.g. use a Plan-Apochromat 10x/0.45, WD = 2.1 mm objective.

***Note:*** For higher framerates one could apply an inverted spinning-disc microscope setup if available.

***Note:*** Depending on the scanning time of the microscope it is important to note that there is a limit how many regions/samples one can image in one session because the desired framerate is a limiting factor. E.g. a framerate of 15 min (one image every 15 min) with a scan time of e.g. 5 min per imaging block/region allows maximum three regions to be imaged in the session.

**CRITICAL:** It is important to record the exact framerate in between acquired time-lapse images. This can be done in retrospect by determining the amount of time in between recorded images: e.g. right click on first created image from one time-lapse file and note the time the file was created and do the same for the next consecutive image of the same time-lapse file. The time in between the two images determines the framerate (e.g. 15 min). The framerate is important to know as it determines the units in which the dynamics parameters (Velocity in m/s etc.) are calculated in the further analysis.

***Note:*** Brain slices can be imaged without the PeCon FoilCover, but it will increase the risk of the media drying out and thereby causing unwanted drift of the sample.

Methods video S1. MADM-11 *Emx1-*labeled brain tissue at timepoint E16, not yet Undrifted Note the slight drift in the video, related to step 34.

Methods video S2. MADM-11 *Emx1-*labeled brain tissue at timepoint E16, Undrifted and almost no drift in the video, related to step 34

Methods video S3. MADM-11 *Emx1-*labeled brain tissue at timepoint E14, related to step 52

Methods video S4. MADM-5 *Emx1-*labeled brain tissue at timepoint E16 Note the developing axons of the neurons finding their way in the intermediate zone, related to step 52.

### Correction of non-linear local drift in time-lapse images

**Timing: 1–5 h depending on the processing power of the computer**

Here we describe the application of ‘Undrift’, a recently developed tool^1^ to correct non-linear local drift in recorded time-lapse images available at http://github.com/hippenmeyerlab/undrift (https://doi.org/10.5281/zenodo.10183022). Undrift is based on a python script that can be executed by using the Anaconda python platform. Unlike other drift correction options, such as current ImageJ plugins, Undrift facilitates the automatic application of local drift correction to time-lapse images containing two fluorophores. The text highlighted in gray are commands to be typed in the Anaconda terminal. See Methods video S1: MADM-11 *Emx1-*labeled brain tissue at time point E16, not yet Undrifted and Methods video S2: MADM-11 *Emx1-*labeled brain tissue at time point E16, Undrifted for an example of before and after Undrift application related to Step 34.

Exporting and processing images.

32. Apply an orthogonal projection of the time-lapse z-stack for each time-lapse video or keep as a z-stack depending on the need for further analysis.
33. Export time-lapse images as ∗.tiff file for each imaging block/time-lapse (e.g., filename_time-lapse.tiff).
34. Apply Undrift (https://doi.org/10.5281/zenodo.10183022) to reduce potential sample drift (See http://github.com/hippenmeyerlab/undrift for details and examples).
  a. Installation.
    i. Download and install the python platform Anaconda (e.g., in C:\Users\username\Anaconda3)).
    ii. Run Anaconda and install git by running the command: >conda install git.
    iii. Clone repository to <path> (e.g., the path where you want the python repository to be stored). For installing in the directory you are in use the command:
      >git clonehttps://github.com/hippenmeyerlab/undrift
    iv. Move to the directory where Undrift were installed:>cd undrift
    v. Install required packages by running the command:
      >pip install -r requirements.txt -e .
  b. Use Undrift.
    i. Basic command to apply Undrift to time-lapse images:
      >undrift <time-lapse file>
      E.g. >undrift C:\time-lapse-images\filename_time-lapse.tiff
    ii. It is possible to change to change the spatial smoothing of Undrift (standard is set to 31 pixels).
      To use undrift with spatial smoothing of e.g., 51 pixels use command.
      >undrift C:\time-lapse-images\filename_time-lapse.tiff --smooth_xy 51
    iii. The output will generate three new files:
      filename_drift_visu.tiff (visualization of the image drift in a checkboard style, with fields corresponding to the smooth_xy pixel value), filename_optflow_field.tiff (Optical flow field estimation of the image) and filename_undrift.tiff (final corrected image file).
    iv. Check if the time-lapse images (filename_undrift.tiff) have been corrected. If not, run Undrift again with different smooth_xy values until optimal drift correction has been achieved.
    v. Apply Undrift with the same parameters to all time-lapse images planned to be used in the same future dataset.
35. Analyze the corrected time-lapse images with the software of choice.

***Note:*** The time-lapse images have to be assembled as an image stack i.e. all individual frames in a single ∗.tiff file to be used with Undrift.

***Note:*** To use Undrift, it is required to install Anaconda, an open-source Python distribution platform.

***Note:*** Most commands described in this protocol can be copied directly into the Anaconda prompt.

***Note:*** The amount of spatial smoothing that Undrift will use for the correction vector field can be adapted to the respective images. E.g. If the time-lapse images have a resolution of 512 × 512 pixels and you want to apply ∼10% smoothing of file pixel size, apply 51 pixels smoothing in Undrift: >undrift C:\time-lapse-images\filename_time-lapse.tiff --smooth_xy 51

For ∼5% smoothing from file a pixel file size of 512 × 512 pixels xy, use 25 pixels).

The smooth function only accepts integers so decimal values are not accepted.

***Note:*** For help with undrift use command:>undrift --help

***Note:*** If you used another path than C:\ to clone the repository, the command is:

>cd <path>/undrift)

***Note:*** Undrift can be applied recursively to many time-lapse images in the same folder e.g. using Windows: >for /r %i in (∗.tif) do undrift --smooth_xy 51 "%i"

***Note:*** In case of extreme drift in the images, it might not be suitable to use such a time-lapse as a correction using Undrift might not be sufficient and therefore proper analysis might not be possible.

***Note:*** At the edges of images with local drift, Undrift processing will remove a part of the image to correct the drift.

***Note:*** If correction of drift is necessary for any recorded time-lapse video, it is important to apply the drift correction in all time-lapse images used for later analyses to avoid any possible correction bias.

### Analysis of neuronal trajectories

**Timing: the automatic tracking is fast (1–5 min) whereas manual curation and correction of tracks can take longer (30 min** to **2 h per video).**

Here we will describe an example of how one can analyze migrating neurons in time-lapse images by using ImageJ (FIJI) and the TrackMate plugin semiautomatically. For further details refer to.^1^

36. Open ImageJ^9^ and load the desired time-lapse file to be analyzed (File *→ Open* or simple drag and drop your file into ImageJ).
37. Go to *Image → Properties* and set the Frame interval to the respective framerate at which the time-lapse was recorded (e.g., 15 min) and set the Pixel width and Pixel height to the corresponding values in micrometer (e.g., pixel width and height 1.2479 μm and Voxel depth 5 μm).
38. Save the file while keeping it open in ImageJ (*File → Save → Replace*).
39. Start the plugin TrackMate^10^ (*ImageJ (FIJI) → Plugins → Tracking → TrackMate* ).
40. Check that the Calibration and Crop settings are set correctly for the loaded file.
41. Choose the LoG detector and press next.
42. Choose the channel to be analyzed (Segment in channel) and set Estimated blob diameter: 10.0 μm, threshold: 2.0, Median filter: enabled, Sub-pixel localization: enabled. Press next.
43. After detection, press next.
44. Choose Auto in Initial thresholding and press next.
45. In select a view, Choose Hyperstack displayer and press next.
46. In the next window one can apply filters on spots if needed and press next.
47. In “Select a tracker” choose Linear motion LAP tracker and set values (Initial search radius: 15, Search radius: 15, Max frame gap: 2).
48. Curate each track manually to ensure that the tracking was successful. Either correct the tracking manually or discard track using the function “TrackScheme”.
49. Save the TrackMate file by Clicking “Save”.
50. Extract all parameters by clicking “Analysis” and three windows with analysis data will pop-up. Save the data as a .csv file by pressing *File → Save in each pop-up window.*
51. Repeat the TrackMate analysis for the other channel in the image by restarting the process.
52. Analyze extracted data as desired by e.g., calculating velocity, directionality etc. (See expected outcomes).

***Note:*** Depending on the goal of the analysis, it can be important to distinguish double-labeled cells (yellow) from purely red and green labeled cells. Yellow cells can be filtered by setting filters on detected spots in TrackMate for e.g. Median intensity to be above or below a certain value for each analyzed image channel to filter out only red or green labeled cells.

## Expected outcomes

The expected outcome should be similar to that observed in Methods videos S1, S2, S3, and S4, a time-lapse of migrating neurons. Depending on the quality of the recorded images, one can use the Undrift tool as described above, to attain videos that can be analyzed with minimal distortion (e.g., tissue drift).

One can proceed with the image analysis to track the migrating neurons over time when the time-lapse of neurons has been obtained. Once neurons have been tracked they can be analyzed on different parameters such as e.g., speed, directionality, track distance, acceleration, etc. In Hansen et al. 2022, we analyzed several parameters of the neuronal migration dynamics of projection neurons in the developing mouse cortex. The neuronal migration tracking data from the time-lapse imaging experiments also served as the basis for a quantitative modeling approach to neuronal migration.^1^

## Quantification and statistical analysis

For the tracking data, a range of parameters can be analyzed for describing the dynamics of neuronal migration as found below:

Distance of one cell between two frames as:

$$d\left(x_{i},x_{j}\right)\sqrt{\sum_{d=1}^{2}{\left(x_{i,d}-x_{j,d}\right)}^{2}}$$

Total distance traveled:

$$d_{tot}=\sum_{i=1}^{N-1}d\left(x_{i},x_{i+1}\right)$$

Net distance traveled:

$$d_{net}=d\left(x_{1,}x_{N}\right)$$

Net time traveled:

$$t_{net}=t_{n}-t_{1}$$

Mean straight line speed:

$$d_{net}/t_{net}$$

Directionality (meandering) index:

$$d_{net}/d_{tot}$$

The tools to analyze the tracked neurons could e.g., be R Studio or Excel and the final statistical analysis can e.g., be carried out with GraphPad Prism or similar software.

## Limitations

Our protocol describes time-lapse imaging of passively cultured brain tissue that allows the analysis of the neuronal dynamics during mouse brain development. Tissue can be cultured and imaged for many hours (>24 hours, See Methods video S4: MADM-5 *Emx1-*labeled brain tissue at time point E16 for an example), however, over extended periods (>12 hours) the tissue can start to disintegrate and be less viable. Depending on the goal of the experiment, one can adjust the time needed to record the biological process of interest.

## Troubleshooting

### Problem 1

The genotyping process is long and the tissue needs to be stored longer before imaging can start (related to step 5).

### Potential solution

If a time-consuming genotyping protocol is required, we recommend oxygenating the ice cold ACSF in which the brains are stored to ensure their viability. Alternatively, one can continue with the protocol until step 19 before genotyping and store the cultured slices in the incubator until the genotyping has finished. Of note, long storage of slices before imaging most likely reduces the viability of the tissue. In extreme cases, one may decide to image immediately and genotype in retrospect.

### Problem 2

Tissue does not stick to the embedding low-melting agarose after vibratome slicing and limits careful transfer to the culture-dish with a tweezer (related to step 18).

### Potential solution

Transfer the cut brain slice to the culture dish with a pipette with a large opening.

Alternatively, make sure to dry the brain before embedding it in low-melting agarose.

### Problem 3

Floating specimen (related to step 19).

### Potential solution

Only barely cover the tissue slice with media, do not add to much media, as the specimen will start floating. If too much media was applied, simply remove excess media by pipetting on the side of the filter insert until the slice is harbored on the filter-insert again.

### Problem 4

Tissue disintegration (related to step 20).

### Potential solution

This problem is hard to avoid and the best way to keep the tissue intact is to minimize the handling of the tissue slice until it is placed on the filter insert and ready for imaging. Proper embedding in the low-melting agarose can help minimizing tissue disintegration and slicing no thicker than 400 microns. We advise to image multiple slices from the same animal simultaneously to increase the chances of useful images. It can be done by culturing several slices on the same filter insert or using 6-well glass-bottom dishes instead of just one single glass-bottom dish.

### Problem 5

Viability of cells in brain slices (related to step 20).

### Potential solution

When using MADM, the fluorescent markers are expressed endogenously and therefore require a living cell to be expressed. Hence, dead cells would not be able express the fluorescent markers. It is challenging to estimate the viability of brain slices without fixing and staining the tissue. One possibility could be to perform a post-imaging fixation and apply a live/death staining using Propidium Iodide (PI) to assess the viability of the brain tissue slice.^11^ However, staining and imaging thick tissue in order to identify single cells can be challenging, but could provide a general overview of the viability of the tissue slices. Dying cells can be identified by dimmer fluorescence, halted migration and most often displaying fragmented processes and/or vesicular inclusions in the cell body.

In the authors’ experience, neonatal mouse brain tissue slices can stay viable for a few hours in ice-cold ACSF. In culture media, brain slices can stay viable for long periods of time (>24 hours, See Methods video S4: MADM-5 *Emx1-*labeled brain tissue at time point E16 for an example of a long video). Longer storage would require a more substantial nutritional support of culture media. Long-term imaging of brain tissue slices from more mature animals (>postnatal day 7) may require continuous perfusion of fresh culturing media. To ensure that each batch is comparable, we recommend parallel imaging of several brain slices with the genotype of interest, including their respective controls.

## Resource availability

### Lead contact

Further information and requests for resources and reagents should be directed to and will be fulfilled by the lead contact Simon Hippenmeyer (simon.hippenmeyer@ist.ac.at).

### Technical contact

Additional requests regarding technical details should be directed to the technical contact, Andi Harley Hansen (andiharley@gmail.com).

### Materials availability

This study did not generate new unique reagents.

### Data and code availability

All scripts that were used to prepare data and figures for this manuscript are available via GitHub at http://github.com/hippenmeyerlab. GitHub DOI is listed in the key resources table. Any additional information required to reanalyze the data reported in this paper is available from the lead contact upon request.
